# Genetic divergence in Cork Oak based on cpDNA sequence data

**DOI:** 10.1186/1753-6561-5-S7-P13

**Published:** 2011-09-13

**Authors:** Joana Costa, Célia Miguel, Helena Almeida, Margarida M Oliveira, José A Matos, Fernanda Simões, Manuela Veloso, Pinto C Ricardo, Octávio S Paulo, Dora Batista

**Affiliations:** 1 Faculdade de Ciências da Universidade de Lisboa/Centro de Biologia Ambiental (CBA), Computational Biology and Population Genomics Group (http://cobig2.fc.ul.pt/), Edifício C2, Campus da Faculdade de Ciências, 1759-016 Lisboa, Portugal; 2 Instituto de Tecnologia Química e Biológica, Universidade Nova de Lisboa (ITQB-UNL)/Instituto de Biologia Experimental e Tecnológica (IBET), Av. da República, 2780-501 Oeiras, Portugal; 3 Universidade Técnica de Lisboa, Instituto Superior de Agronomia (ISA), Centro de Estudos Florestais, Tapada da Ajuda, 1349-017 Lisboa, Portugal; 4 Instituto Nacional de Recursos Biológicos, I.P. (INRB), L-INIA - Pólo do Lumiar, Unidade Investigação Recursos Genéticos, Ecofisiologia e Melhoramento de Plantas, Grupo Biologia Molecular, Estrada Paço Lumiar 22, Ed.S 1°, 1649-038 Lisboa, Portugal; 5 INRB, I.P., L-INIA - Pólo de Oeiras, Unidade Recursos Genéticos, Ecofisiologia e Melhoramento de Plantas, Quinta do Marquês, 2784-505 Oeiras, Portugal; 6 Instituto de Investigação Científica Tropical IICT/CIFC, Quinta do Marquês, 2784-505 Oeiras, Portugal

## Background and objectives

Cork oak (*Quercus suber* L.) is one of the dominant broadleaved woody species in the western Mediterranean Basin, defining unique open woods. These woodlands have an outstanding economical and ecological value in this region, particularly in Portugal, where they sustain a strong cork industry. In the context of a prospective management of these sustainable ecosystems and renowned reservoirs of biodiversity, it is vital to better understand how the genetic variation of *Q. suber* natural populations is spatially organized so reasonable guidelines for conservation can be provided. On the other hand, knowledge of how past climate fluctuations influenced the patterns and dynamics of *Q. suber*, shaping the ranges of the species in the Mediterranean peninsulas, is of the utmost importance for our perception of what can happen in the future. Although a great deal of details on the genetic divergence of the Mediterranean cork oak populations has been uncovered and several hypotheses have been advanced concerning its evolutionary history, there is still much to unravel. For instance, Portuguese natural population sampling included so far in previous studies has been very deficient. To achieve this goal a different and complementary analysis of cork oakÂ´s genetic diversity was initiated under a phylogeographical framework based on chloroplastidial DNA sequences. This study is the starting up of a project aiming at assessing the genetic diversity and differentiation of natural cork oak populations from the entire Mediterranean distribution, with the intent of understanding patterns of biodiversity, gene flow and population admixture, as well as to infer possible evolutionary events.

## Materials and methods

We used 3-5 samples from 25 populations collected across the range of distribution of cork oak, in a total of 115 individuals. Three chloroplastidial DNA (cpDNA) intergenic spacer regions (*TrnL-F*[1], *TrnS-PsbC*[2] and *TrnH-psbA*[3]) were amplified and sequenced. Fourteen individuals of *Q. rotundifolia*, five of *Q. coccifera*, two of *Q. ilex*, and one of *Q. robur*, *Q. faginea*, *Q. pyrenaica*, *Q. lusitanicus*, *Q. rubra* and *Q. canariensis* were also analyzed as comparative references. *Castanea crenata* was used as outgroup. The alignment was developed in Clustal X 2.0.12 [4] and manually refined. The phylogenetic trees were made with PAUP 4.0d99 [5], using a Maximum Parsimony analysis method.

## Results

Regarding the cpDNA regions under study, two main lineages of cork oak haplotypes were found: one lineage closely related to *Quercus rotundifolia* and *Q.coccifera*, (“introgressed lineage”), mainly present in Iberia and Morocco; and a second lineage that contrastingly seems “pure”, which is the most common and does not appear to be shared with any other *Quercus* species (“pure lineage”). Three distinct sublineages are shown in the “pure” lineage, corresponding to the Eastern populations, Sicilly and the Western populations, although separated by minor differences. Some populations present cpDNA haplotypes belonging to both lineages, while others are specific for one of them. An absence of introgression in the easternmost populations of cork oak seems also apparent.

## Conclusions

Although these preliminary results seem to confirm previous ones, some major differences are suggested, such as too few differences between the western and eastern groups to be explained by a Tertiary divergence pattern, as previously suggested [6], but rather more consistent with a more recent expansion from few refugia; and widespread and multiple introgression events from *Q. rotundifolia* and *Q. coccifera*, common in peripheral western populations.
